# Cultural Variation in Reactions to a Group Member’s Vicarious Choice and the Role of Rejection Avoidance

**DOI:** 10.3389/fpsyg.2019.01311

**Published:** 2019-06-07

**Authors:** Charis Eisen, Keiko Ishii

**Affiliations:** ^1^ Department of Social Sciences, Darmstadt University of Applied Sciences, Darmstadt, Germany; ^2^ Department of Psychology, Graduate School of Informatics, Nagoya University, Nagoya, Japan

**Keywords:** agency, culture, rejection avoidance, personal choice, vicarious choice

## Abstract

Extending the literature on culture and the personal or interpersonal construction of choices, this research investigates consequences of an ingroup member’s vicarious decision for the entire group and the mechanism behind cultural variation. In Study 1, Japanese people showed, compared to Germans, greater acceptance of vicarious choice and evaluated the ingroup member who had chosen on their behalf more positively. Using mediation analyses and priming methods, Studies 2 and 3 identified rejection avoidance to partly explain culturally diverse reactions to vicarious choices. These findings suggest that the mechanism behind cultural differences in choice is related to variation in strength of the motivation to maintain social approval.

“It is our choices, Harry, that show what we truly are” (Harry Potter and the Chamber of Secrets, 1998). Rowling’s (1998) line reveals how central choice is for an individual’s psychology and life course. The provision of choice and self-determination are crucial for autonomy and human motivation and make individuals happier and healthier (Zuckerman et al., 1978; Deci and Ryan, 2008). On the other hand, evidence on social influence suggests that individuals tend to adjust themselves to the thought of the majority in a group pressure situation (Asch, 1952; Cialdini and Goldstein, 2004). Although both the pursuit of personal choice and seeking to fit in the group underlie individuals’ behaviors, which of these two aspects is emphasized might be moderated by cultural differences in the weight on self and social relationships. Indeed, a growing research stream has documented that Western individualistic cultures generally promote a stronger desire for personal choice, but a smaller influence of interpersonal concerns on decisions than East Asian collectivistic cultures (e.g., Kitayama et al., 2004; Savani et al., 2008, 2010).

In this research, drawing on the literature on culture and choice, we examine cultural differences in reactions to a group member’s vicarious choice in Germans and Japanese. Based on previous work suggesting that East Asians are more motivated than Westerners to avoid rejection by group members (Sato et al., 2014; Hashimoto and Yamagishi, 2016), we hypothesized that Japanese would be more likely than Germans to react positively to vicarious choice and that cultural differences in rejection avoidance would account for the cultural influence on response to vicarious choice.

## Vicarious Choice

The self has been featured as being independent and separate from other people in Western cultures such as in Germany, whereas it has been featured as being interdependent and connected with others in East Asian cultures such as in Japan (Markus and Kitayama, 1991). Against the backdrop of the greater emphasis on independence, the pursuit of personal choice is crucial for Westerners, as choice enables them to express their individual, autonomous selves through showing their preferences, attitudes, values, and feelings (Kim and Sherman, 2007). On the other hand, Eastern cultures place greater emphasis on social adjustment and accommodation to others (Morling et al., 2002), while self-expression through choice is relatively unimportant.

Cultures promote different implicit frameworks of normative behavior, called models of agency (Markus and Kitayama, 2003). North American and Western European contexts promote a rather disjoint model of agency, which characterizes good actions by their independence of social circumstances and their contingency on one’s own preferences, goals, intentions, and motives. On the contrary, many East Asian contexts promote a rather conjoint model of agency, in which actions are responsive to others’ obligations and expectations, and good actions promote interdependence with and adjustment to others (Markus and Kitayama, 2003; Kitayama and Uchida, 2005; Markus et al., 2006).

Mirroring these divergent models of agency, previous research has illustrated sociocultural variation in emphases on personal and interpersonal aspects in choice. Research identified cultural differences in whether choice is considered individual, personal decision-making or whether multiple people can be involved in a more interpersonally constructed form of choice (Markus and Kitayama, 2003; Mesquita and Markus, 2004; Savani et al., 2010). People in many cultures base their choices not merely on their personal preferences, but rather seek advice and include others’ opinions in their choices without feeling constricted or burdened (Savani et al., 2008, 2010; Eisen et al., 2016). Consistently, previous research illustrated that whereas Asians and Asian Canadians showed no cognitive dissonance in a condition with the standard free-choice paradigm, dissonance was observable in a condition where interpersonal concerns and worries were induced by presenting eyes of others (Kitayama et al., 2004) and when they were to make a choice for their friends (Hoshino-Browne et al., 2005).

Although evidence is limited, the consequence of the denial of choice also differs across cultures. For instance, in Savani et al.’s (2008) Study 5, the experimenter usurped participants of their personal pen choices by choosing on their behalf. The researchers found that, compared to an own choice condition, North Americans indicated less liking of the pen in the usurped choice condition, while Indian participants rated the pens equally likable in both conditions. In addition, Jonas et al. (2009) asked participants to imagine that a colleague they briefly knew requests the abandonment of a certain good participants assumed as theirs, or that an authority prohibited certain products for health reasons, and measured self-reported negative reactions to these scenarios. They found that compared to people from collectivistic cultures, people from individualistic cultures showed greater psychological reactance when they had to give up their personal freedom to use the respective good. As described in reactance theory (Brehm, 1966), the denial of personal choice by another person’s vicarious choice likely threatens Westerners’ freedom and therefore elicits negative responses (reactance) from them. Moreover, Jonas et al. (2009) manipulated independent and interdependent orientations and found that people primed with independent orientation reported more reactance than did those primed with interdependent orientation when they had to give up personal freedom.

In this research, we pay attention to situations in which someone else decides on behalf of a group, for example, when selecting or saying something on the others’ behalf, and thereby restricts the others in their expression of personal preferences and ideas. Studies have examined how choices made by ingroup (e.g., mother) or outgroup (e.g., experimenter) choosers on behalf of the individual affect this individual’s performance and judgments (Iyengar and Lepper, 1999; Hoshino-Browne et al., 2005; Savani et al., 2008). However, vicarious choice situations in which an equal-status ingroup member decides on behalf of the entire group have not received sufficient attention – in spite of being frequent daily life occurrences. Examinations of the consequences of vicarious choices made for the whole group could add to the understanding of how agency is understood in interdependent contexts. Therefore, the present research focuses on these vicarious choices and investigates reactions in Eastern and Western cultures.

## The Role of Rejection Avoidance in the Response to Vicarious Choice

While negative reactions to the denial of freedom are associated with independence of the self (Savani et al., 2008; Jonas et al., 2009), a positive reaction to the denial of freedom might be associated with interdependence of the self. Although this possibility has been suggested by Iyengar and Lepper (1999), who found an association between personal choice and intrinsic motivation in European American children and an association between a choice made by a close other (e.g., their mothers) and intrinsic motivation in Asian American children, the underlying psychological mechanism of such a cultural difference has not been fully tested.

To explore the underlying mechanism of a cultural difference in the response to vicarious choice, we focus on how being afraid of social rejection leads to avoidant behavior. People care deeply about social rejection, as they want to connect with other people in their own group. The need to belong has been shown to play a significant role across cultures (Fiske and Yamamoto, 2005). Previous research illustrated that experience of rejection leads to rejection sensitivity, which in turn promotes rejection avoidance behaviors (e.g., Feldman and Downey, 1994). Molden et al. (2009) showed that when people recalled or underwent experiences of being rejected, they showed prevention-focused responses. Similarly, studies suggest that rejection experiences bring to mind broader social connections, for example, social groups one belongs to (Knowles and Gardner, 2008) and promotes to seek out group settings (Maner et al., 2007), to adhere to group norms (Kerr et al., 2009), and to increase contribution to group efforts (Williams and Sommer, 1997). Taken together, these findings suggest that the experience of being rejected generally leads to increases of rejection sensitivity and avoidance at the individual level.

However, the literature also suggests cultural differences in the significance of the strategy to avoid rejection in order to live a good life. Hashimoto and Yamagishi (2013, 2016) argued that a society like Japan, which is maintained by mutual monitoring and sanctioning within fixed group boundaries, promotes heavy dependency of individuals on others. As groups are closed to outsiders and mutual commitment relationships are prevalent, rejection by group members and exclusion from the community-based cooperation system is very harmful. It is therefore wise to be sensitive to the needs and expectations of other members of the group and not to offend them in order to avoid social rejection. In contrast, a society like Germany allows its members to find alternative interaction partners easily, and therefore, social rejection is not as deleterious. Sato et al. (2014) have provided empirical evidence for the claim that being rejected is more threatening to East Asian people than for Westerners because the cost of being disliked and eventually excluded by others is greater in these societies, where finding alternative relationships is rather difficult. Other studies have consistently found that East Asians exhibit more pronounced rejection avoidance tendencies than Westerners (Yamaguchi et al., 1995; Garris et al., 2011). Consistently, empirical research has shown that Asians oftentimes behave in a way that allows them to avoid any disruption of harmonious relationships: Compared to Westerners, East Asians more frequently engage in self-criticism (Heine et al., 2000), are less willing to seek social support (Kim et al., 2008; Ishii et al., 2017), and more frequently inhibit their desire to express disagreement (Hashimoto et al., 2012) in order to prevent social disapproval. These findings suggest that although rejection poses a threat and experiences of rejection lead to avoidance behaviors to people regardless of their cultural background, structural factors (e.g., whether a society is maintained by a mutual monitoring and sanctioning system) promote these avoidance behaviors to varying degrees.

The concern for others’ appraisals might lead individuals across cultures to feel a threat of rejection from the group they belong to, particularly in situations in which all group members form a mutual commitment relationship and can observe individuals’ behavior. Choosing based on one’s inner attributes while ignoring the social context or failing to incorporate others’ preferences could be seen as incongruent to social standards and thus potentially elicit rejection by the other group members. However, as structural factors in Japan promote the prevention of social rejection more strongly than structural factors in Germany, Japanese people might respond more positively to vicarious choice by ingroup members, while Germans would be more likely to risk rejection and claim personal choice. We hypothesized, accordingly, that the cultural differences in reactions can be partly explained by variation in levels of rejection avoidance.

## The Present Research

The present research conducted three studies among German (representing a Western culture) and Japanese (representing an East Asian culture) participants to explore cultural differences in responses to vicarious choice. We hypothesized that Germans would be more likely than Japanese to demand choice as a reaction to vicarious choice and evaluate the chooser more negatively, whereas Japanese would be more likely than Germans to accept vicarious choices and evaluate the chooser more positively. Following Study 1, which tests the cultural differences, Studies 2 and 3 hypothesized that Japanese would be higher in rejection avoidance than Germans and that higher rejection avoidance would lead people to accept vicarious choice and demand less personal choice.

Given that rejection avoidance reflects a concern for social disapproval, the impact on responses to vicarious choice would be clearly demonstrated in a group situation such as when people are working in a team. Thus, we developed a set of group scenarios that one group member first chooses and proposes a collective behavior without asking and considering individual preferences and opinions. Little is known about cultural differences in reactions to vicarious choice in a group setting, as previous findings that the consequences of vicarious choice depend on culture are mainly based on the examinations at the dyadic level (e.g., mother; Iyengar and Lepper, 1999). Testing with these group scenarios, we also explore the cultural differences in reactions to vicarious choice can be generalized even in a condition where group pressure can be estimated and perceived.

## Study 1A

### Method

#### Ethics Statement

The study was reviewed and approved by the Experimental Research Ethics Committee at the Graduate School of Humanities, Kobe University. Participants provided a written informed consent at the beginning of the study. All responses were confidential.

#### Participants

One hundred and ten Japanese (55 women, *M*_age_ = 44.9, SD_age_ = 14.35) and 99 German adults (50 women, *M*_age_ = 43.0, SD_age_ = 13.95) were recruited through an online crowdsourcing service in each country (Macromill in Japan, Respondi AG in Germany). The participants were paid according to local standards. We determined the sample size by referring to Jonas et al. (2009, Study 1), showing a small/medium effect size for the main effect of culture (*d*s = 0.33–0.61). We estimated that the sample size was 99 for each culture, assuming the effect size (*d*) as 0.4 and a value for desired power as 0.80.

#### Procedure

We composed a questionnaire in Japanese and translated it into German. A bilingual third-party back-translated the questionnaire into Japanese, and we compared this back-translation to the original to assure that all questions were equal in meaning. We asked participants to imagine the following three scenarios:

1. You finished a big project at work and your colleagues and you go to celebrate this success with a business meal. At the restaurant, you find a menu, but one of your colleagues orders for the whole team without asking for individual preferences.
2. You plan an event together with your coworkers, and there are many tasks to share. Someone needs to take care of the finances, someone needs to do advertising, someone needs to invite and take care of the guests, and someone needs to do the paper work. One of your coworkers takes the lead and tells you and the others what to do without asking for individual preferences.
3. You are in a meeting and your boss asks for feedback about a new policy that he introduced last week. One of your colleagues answers in detail, representing the whole team without asking individual opinions.

For each scenario, participants indicated on three items how likely they would be to accept the vicarious choice (e.g., “accept the decision of the colleague”) and on two items how likely they would be to demand personal choice (e.g., “choose something to eat and drink from the menu yourself”) on Likert-type scales ranging from 1 (*very unlikely*) to 7 (*very likely*), respectively. In the following analyses, we created an index for acceptance and an index for choice demand over all three scenarios. However, the results were the same when we analyzed them separately.

Furthermore, participants indicated how positively (i.e., *likable* and *sociable*) and negatively (i.e., *egoistic* and *dominant*)^1^ they would evaluate the ingroup member who had chosen for the entire group in such a situation on 7-point scales (1 = *strongly disagree*, 7 = *strongly agree*). Moreover, participants indicated their agreement to the statement “a situation like this occurs frequently” on a 7-point scale (1 = *strongly disagree*, 7 = *strongly agree*) for each scenario.

### Results

#### Frequency

If vicarious choices are, as expected, more positively connoted in Japan as compared to Germany, it would be natural if vicarious choice situations were more frequent occurrences in Japan than in Germany. Indeed, across scenarios (*α*_Ger_ = 0.82, *α*_Jap_ = 0.76), Japanese participants (*M* = 4.30, SD = 1.10) indicated that such situations are frequent daily life situations more than German participants (*M* = 3.57, SD = 1.61, *t*(207) = 3.86, *p* < 0.001, *d* = 0.53, 95% CI = [0.36, 1.10]). However, as we were interested in consequences of vicarious choice situations independent of how frequent such situations occur, we controlled for this variable in the following analyses^2^.

#### Reaction

**Figure 1:**
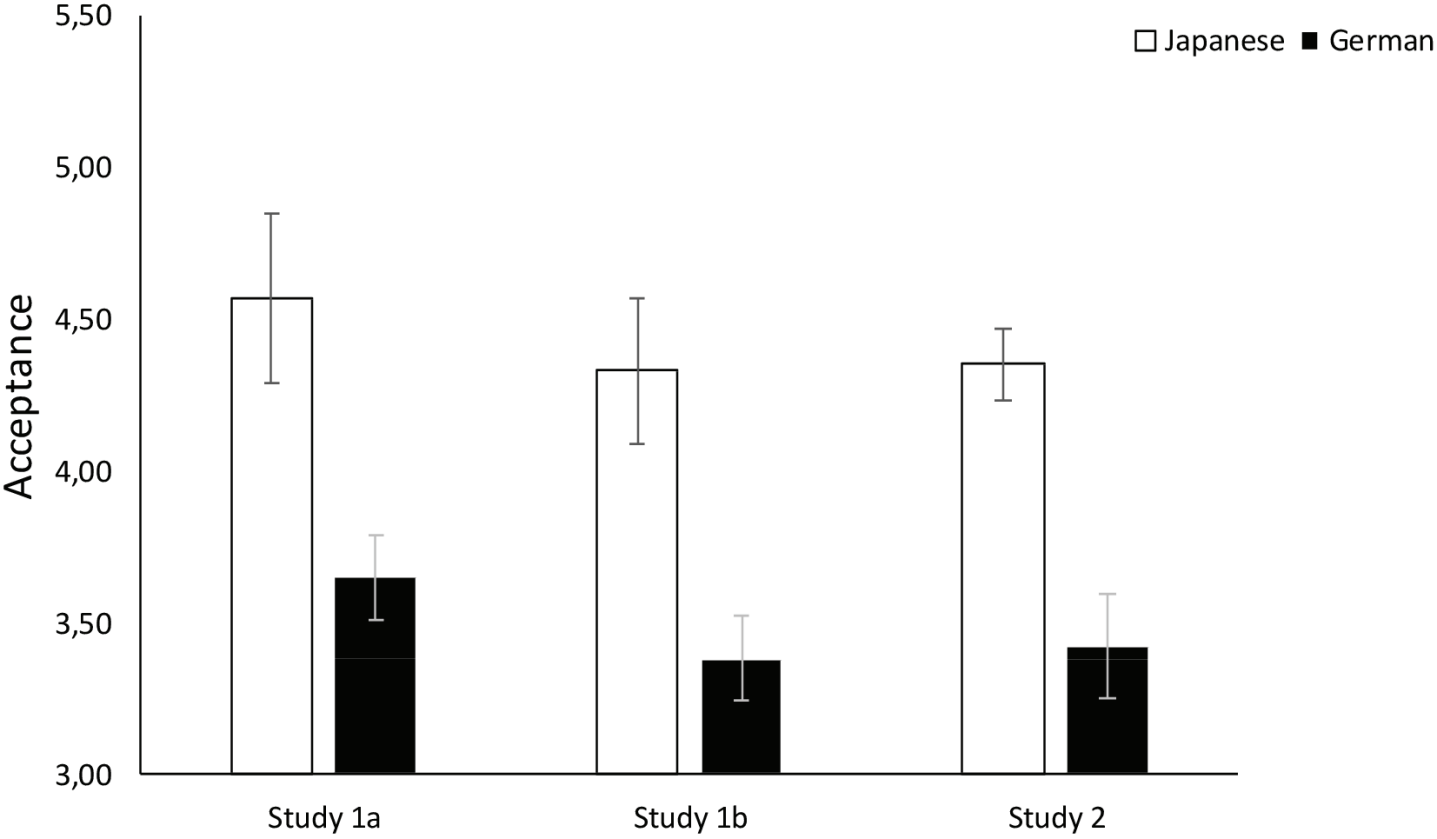
Cultural differences in acceptance of vicarious choice in Studies 1a, 1b, and 2. Error bars show 95% confidence intervals.

**Figure 2:**
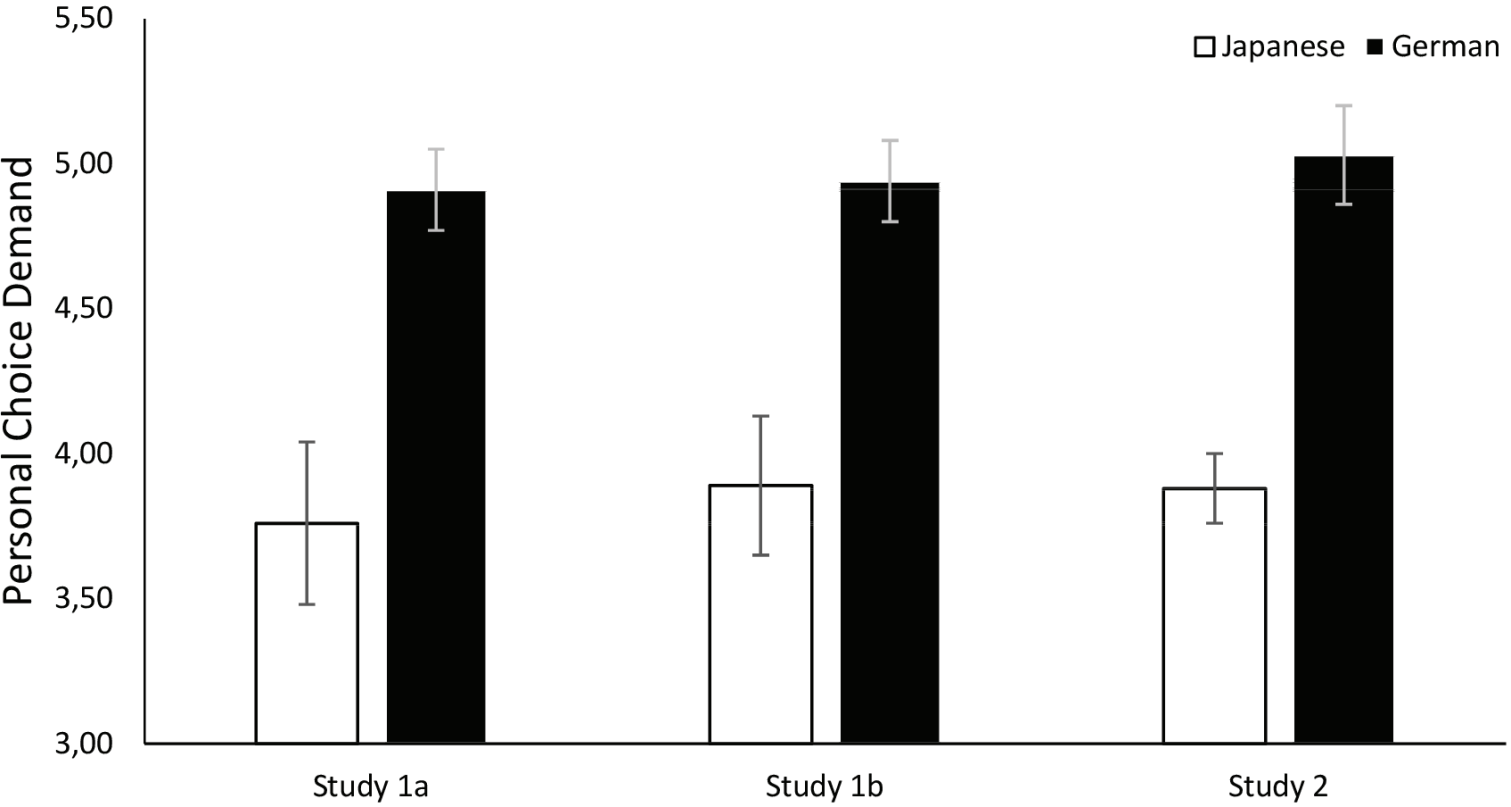
Cultural differences in personal choice demand as a reaction to vicarious choice in Studies 1a, 1b, and 2. Error bars show 95% confidence intervals.

We calculated participants’ likeliness to accept the vicarious choice (*α*_Ger_ = 0.87, *α*_Jap_ = 0.84) and demand personal choice (*α*_Ger_ = 0.74, *α*_Jap_ = 0.71) in all three scenarios and conducted ANCOVAs to investigate cultural differences. As predicted, Japanese participants (*M* = 4.57, SD = 0.97) were significantly more likely than German participants (*M* = 3.65, SD = 1.29) to accept the choices on their behalf: *F*(1, 206) = 24.03, *p* < 0.001, ${\eta^{2}}_{p}$ = 0.10. In addition, German participants (*M* = 4.91, SD = 1.18) were significantly more likely than Japanese participants (*M* = 3.76, SD = 0.97) to demand personal choice: *F*(1, 206) = 57.74, *p* < 0.001, ${\eta^{2}}_{p}$ = 0.22. The results are illustrated in Figures 1, 2.

#### Evaluation

We calculated how positively (*α*_Ger_ = 0.84, *α*_Jap_ = 0.68) and negatively (*α*_Ger_ = 0.83, *α*_Jap_ = 0.80) the participants would evaluate the ingroup member who had chosen for the entire group across scenarios and compared German and Japanese respondents. As predicted, Japanese evaluated the chooser as more likable and sociable (*M* = 3.77, SD = 0.80) and less egoistic and dominant (*M* = 4.42, SD = 1.01) than Germans did [positive: *M* = 3.04, SD = 1.23, *F*(1, 206) = 15.82, *p* < 0.001, ${\eta^{2}}_{p}$ = 0.07; negative: *M* = 5.27, SD = 1.23, *F*(1, 206) = 28.94, *p* < 0.001, ${\eta^{2}}_{p}$ = 0.12].

## Study 1B

In Study 1a, we constructed the scenarios such that the person choosing for the entire group is a colleague and not an individual who is higher or lower in the social hierarchy. However, we did not make the equal status explicit and participants might have assumed that the chooser is someone of higher social status. Therefore, we conducted an additional study to eliminate the possibility that cultural differences are side effects of differences in perceived social status.

### Method

#### Ethics Statement

The study was reviewed and approved by the Experimental Research Ethics Committee at the Graduate School of Humanities, Kobe University. Participants provided a written informed consent at the beginning of the study. All responses were confidential.

#### Participants

One hundred and ten Japanese (55 women, *M*_age_ = 44.5, SD_age_ = 13.96) and 100 German adults (50 women, *M*_age_ = 46.4, SD_age_ = 15.15) were recruited through online crowdsourcing services (as in Study 1a).

#### Procedure

We asked participants to imagine the same three vicarious choice scenarios as in Study 1a, but added the information that the colleagues were all of the same status. We included an item that asked participants to indicate how strongly they agree to the statement “the colleague, who chose for the group, is high in rank” (1 = *strongly disagree*, 7 = *strongly agree*).

As in Study 1a, participants indicated how likely they would be to accept the vicarious choice/demand personal choice and how positively/negatively they would evaluate the ingroup member who had chosen for the entire group.

### Results

#### Perceived Rank

Across scenarios (*α*_Ger_ = 0.87, *α*_Jap_ = 0.65), Japanese participants (*M* = 3.98, SD = 1.00) and German participants (*M* = 3.69, SD = 1.51) perceived the rank of the chooser similarly, *t*(208) = 1.67, *p* = 0.10, 95% CI = (−0.64, 0.05). Thus, the participants in both cultures understood the information of rank correctly, as we intended. In contrast, perceived rank correlated with reactions to vicarious choice (German sample: acceptance *r*(98) = 0.53, *p* < 0.001; choice demand *r*(98) = −0.19, *p* = 0.051. Japanese sample: acceptance *r*(108) = 0.20, *p* = 0.035; choice demand *r*(108) = −0.05, *p* = 0.629). To preclude the possibility that perceptions of rank influenced participants’ reactions, we statistically controlled for this variable in the following analyses^3^.

#### Reaction

We calculated participants’ likeliness to accept vicarious choice (*α*_Ger_ = 0.87, *α*_Jap_ = 0.83) and demand personal choice (*α*_Ger_ = 0.75, *α*_Jap_ = 0.79) and conducted ANCOVAs to investigate cultural differences. As predicted, Japanese participants (*M* = 4.33, SD = 0.93) were significantly more likely than German participants (*M* = 3.38, SD = 1.24) to accept choices on their behalf, *F*(1, 207) = 36.77, *p* < 0.001, ${\eta^{2}}_{p}$ = 0.15, and German participants (*M* = 4.94, SD = 1.13) were significantly more likely than Japanese participants (*M* = 3.89, SD = 1.02) to demand personal choice, *F*(1, 207) = 47.68, *p* < 0.001, ${\eta^{2}}_{p}$ = 0.19. The results are illustrated in Figures 1, 2.

#### Evaluation

We calculated how positively (*α*_Ger_ = 0.89, *α*_Jap_ = 0.85) and negatively (*α*_Ger_ = 0.88, *α*_Jap_ = 0.83) participants would evaluate the ingroup member who had chosen for the group across scenarios. As predicted, Japanese people evaluated the chooser as more likable and sociable (*M* = 3.68, SD = 1.08) and less egoistic and dominant (*M* = 4.71, SD = 1.02) than Germans did [positive: *M* = 3.00, SD = 1.26, *F*(1, 207) = 14.48, *p* < 0.001, ${\eta^{2}}_{p}$ = 0.07; negative: *M* = 5.06, SD = 1.38, *F*(1, 207) = 5.45, *p* = 0.020, ${\eta^{2}}_{p}$ = 0.03].

## Study 2

Study 1 investigated how Germans and Japanese respond to situations in which an ingroup member has made a choice, specifically what to eat, which task to perform, and how to respond, for an entire group. Eliminating possible confounding effects of perceived frequency and social status, the results support the hypothesis that vicarious choice is more accepted and more positively connoted in Japan as compared to Germany. Study 2 aimed at shedding light on the mechanism behind the observed cultural variation. Specifically, we tested whether individual rejection avoidance tendencies mediate cultural differences in reactions to vicarious choices.

### Method

#### Ethics Statement

The study was reviewed and approved by the Experimental Research Ethics Committee at the Graduate School of Humanities, Kobe University. Participants provided a written informed consent at the beginning of the study. All responses were confidential.

#### Participants

Crowdsourcing services recruited two community samples of 220 Japanese (110 women, *M*_age_ = 44.26, SD_age_ = 13.81) and 212 Germans (96 women, *M*_age_ = 43.58, SD*_age_* = 14.08) and reimbursed them according to their standard. We determined the sample size by referring to Jonas et al. (2009, Study 1), showing a small/medium effect size for the main effect of culture, for the expected direct effect. We referred to Hashimoto and Yamagishi (2016), who found a medium effect (*d* = 0.46) for cultural differences in rejection avoidance, for the expected effect of culture on rejection avoidance. Based on Fritz and MacKinnon (2007), we estimated that the sample size should be 404 in total to detect the expected mediated effect using bootstrap analyses with a desired power of 0.80.

#### Procedure

In a questionnaire, we asked participants to imagine the same scenarios as in Study 1 and to indicate their likely reactions to these situations. Thereafter, participants answered a scale measuring rejection avoidance tendencies (Hashimoto and Yamagishi, 2016). The specific items were “I find myself feeling anxious if people are watching me,” “I find myself being concerned about what others think of me,” “I often feel anxious about the nature of my relationships with others and their status as compared to mine,” “I sometimes get so anxious about what other people might think that I am prevented from doing what I really want to do,” and “I often behave in a way that will keep others from disliking me.” Participants indicated how well each of these statements describes them on Likert-type scales (1 = *does not describe me at all*, 7 = *describes me very much*)^4^.

### Results

#### Reaction

We calculated participants’ likeliness of acceptance (*α*_Ger_ = 0.87, *α*_Jap_ = 0.85) and personal choice demand (*α*_Ger_ = 0.76, *α*_Jap_ = 0.72) over all three scenarios. A *t*-test to investigate cultural differences revealed that Japanese participants (*M* = 4.35, SD = 0.97) were significantly more likely than German participants (*M* = 3.42, SD = 1.26) to accept the vicarious choices, *t*(430) = 8.58, *p* < 0.001, *d* = 0.83, 95% CI = (−1.14, −0.71). In addition, German participants (*M* = 5.03, SD = 1.14) were significantly more likely than Japanese participants (*M* = 3.88, SD = 0.97) to demand personal choice, *t*(430) = 11.24, *p* < 0.001, *d* = 1.09, 95% CI = (0.95, 1.35). The results are illustrated in Figures 1, 2. Thus, we replicated the findings of Study 1.

#### Mediation Analyses

To analyze whether individual rejection avoidance tendencies constitute a factor underlying the cultural differences in reactions to vicarious choice, we first calculated rejection avoidance tendencies for each participant by merging the five items measuring this construct (*α*_Ger_ = 0.82, *α*_Jap_ = 0.85). In line with previous research, we found that compared to Germans (*M* = 3.63, SD = 1.33), Japanese (*M* = 4.40, SD = 1.11) were more anxious about being rejected by others, *t*(430) = 6.53, *p* < 0.001, *d* = 0.63, 95% CI = (−1.00, −0.54). Correlation patterns between rejection avoidance and acceptance were *r*_Ger_(210) = 0.45, *p* < 0.001, *r*_Jap_(218) = 0.31, *p* < 0.001 and between rejection avoidance and choice demand *r*_Ger_(210) = −0.36, *p* < 0.001, *r*_Jap_(218) = 0.07, *p* = 0.33.

**Figure 3:**
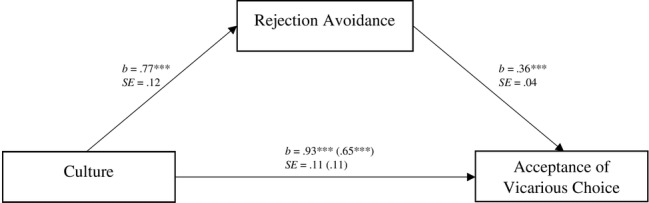
Individual levels of rejection avoidance as a mediator of the cultural differences in acceptance of vicarious choice in Study 2. Unstandardized coefficients and standard errors are shown. Coefficients indicating the relationship between culture (coded as German culture = 0 and Japanese culture = 1) and acceptance of vicarious choice after controlling for rejection avoidance tendencies are given in parentheses. ***p < 0.001.

Next, we conducted mediation analyses to investigate the hypothesis that the cultures provoke different levels of rejection avoidance, which in turn affect reactions to choices on one’s behalf. We dummy coded German culture as 0 and Japanese culture as 1. Regressing culture on acceptance tendencies, we found that culture is a significant predictor, *b* = 0.93, SE = 0.11, *t*(430) = 8.58, *p* < 0.001, 95% CI = [0.72, 1.14]. Culture also predicted rejection avoidance tendencies significantly, *b* = 0.77, SE = 0.12, *t*(430) = 6.53, *p* < 0.001, 95% CI = [0.54, 1.00]. Importantly, individual rejection avoidance tendencies in turn affected reactions to vicarious choice *b* = 0.36, SE = 0.04, *t*(430) = 8.82, *p* < 0.001, 95% CI = (0.28, 0.44) and the predictive power of culture was significantly reduced to *b* = 0.65, SE = 0.11, *t*(430) = 6.23, *p* < 0.001, 95% CI = (0.45, 0.86) when we controlled for rejection avoidance. Bootstrap analyses revealed a significant indirect effect (1,000 bootstrap samples): 95% CI [0.17, 0.41]. Hence, the cultural difference in acceptance of vicarious choice was partially mediated by individual variation in rejection avoidance (Figure 3).

**Figure 4:**
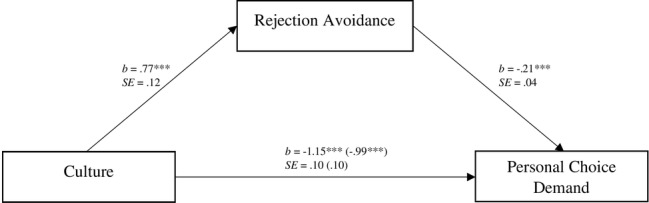
Individual levels of rejection avoidance as a mediator of the cultural differences in personal choice demand as a reaction to vicarious choice in Study 2. Unstandardized coefficients and standard errors are shown. Coefficients indicating the relationship between culture (coded as German culture = 0 and Japanese culture = 1) and acceptance of vicarious choice after controlling for rejection avoidance tendencies are given in parentheses. ***p < 0.001.

Next, we tested whether rejection avoidance tendencies likewise mediated the cultural difference in choice demand. Culture was a significant predictor for choice demand tendencies, *b* = −1.15, SE = 0.10, *t*(430) = −11.24, *p* < 0.001, 95% CI = (−1.35, −0.95). When we entered rejection avoidance simultaneously to an analysis of regression, the effect of culture was significantly reduced, *b* = −0.99, SE = 0.10, *t*(430) = −9.50, *p* < 0.001, 95% CI = (−1.19, −0.78) and rejection avoidance predicted choice demand, *b* = −0.21, SE = 0.04, *t*(430) = −5.05, *p* < 0.001, 95% CI = [−0.28, −0.12]. The indirect effect was significant [1,000 bootstrap samples, 95% CI (−0.26, −0.07)]; suggesting that the cultural difference in choice demand was partially mediated by rejection avoidance (Figure 4).

As an alternative possibility, based on the claim that rejection avoidance is more connected to structural factors in Japan than in Germany, the association between rejection avoidance and responses to vicarious choices could be evident in the Japanese sample, but not in the German sample. We tested this possibility by comparing two regression models, one that includes culture and rejection avoidance to predict acceptance of vicarious choice (model 1) and one that includes also the interaction of culture and rejection avoidance (model 2). Although both models were significant [model 1: *R*^2^ = 0.277, *F*(2, 429) = 82.22, *p* < 0.001; model 2: *R*^2^ = 0.283, *F*(3, 428) = 56.20, *p* < 0.001], the variance explained by these models did not differ significantly [*R*^2^ change = 0.006, *F*(1, 428) = 3.29, *p* = 0.071] and the interaction term was not significant (*b* = 0.15, SE = 0.08, *p* = 0.071). The effect of rejection avoidance was significant in both samples [German sample: *b* = 0.42, SE = 0.05, 95% CI (0.32, 0.52); Japanese sample: *b* = 0.27, SE = 0.06, 95% CI (0.15, 0.40)], suggesting that if someone has a strong tendency to avoid rejection, this person is likely to accept vicarious choice regardless of his/her cultural background. When we performed this analysis with choice demand as dependent variable, both models were significant [model 1: *R*^2^ = 0.270, *F*(2, 429) = 79.53, *p* < 0.001; model 2: *R*^2^ = 0.287, *F*(3, 428) = 59.84, *p* < 0.001]. We found a significant change in *R*^2^ = 0.016, *F*(1, 428) = 9.84, *p* = 0.002 and the interaction term was significant (*b* = −0.25, SE = 0.08, *p* = 0.002). However, quite the contrary to the assumption that rejection avoidance plays a crucial role in Japan but not in Germany, the effect of rejection avoidance was stronger in the German sample (*b* = −0.31, SE = 0.05, 95% CI [−0.42, −0.21]) than in the Japanese sample [*b* = −0.06, SE = 0.06, 95% CI (−0.18, 0.06)]. This indicates that in Germany, people with strong tendencies to avoid rejection would rather not demand personal choice, compared to people with weaker rejection avoidance tendencies. In Japan, people would be unlikely to demand personal choice, regardless of their rejection avoidance tendencies.

## Study 3

Study 2 provided the first evidence to show the mediating role of rejection avoidance in culturally different responses to vicarious choice. Assessing people’s chronic rejection avoidance tendencies, however, Study 2 was based on self-reports and might be compromised by people’s inability to accurately report on their cultural beliefs. To avoid the problems inherent to trait measures and to clarify the causal direction between rejection avoidance and reactions to vicarious choice, we tested in Study 3 whether priming social rejection sensitivity would cause divergent reactions to vicarious choice in Japanese and Germans. Reflecting the importance of belonging across individuals and cultures, previous research illustrated that rejection experiences promote avoidance behavior (Molden et al., 2009). Therefore, we hypothesized that priming rejection sensitivity by asking people to recall a rejection episode would promote rejection avoidant behavior (i.e., acceptance of vicarious choice) regardless of culture.

### Method

#### Ethics Statement

The study was reviewed and approved by the Experimental Research Ethics Committee at the Graduate School of Humanities, Kobe University. Participants provided a written informed consent at the beginning of the study. All responses were confidential.

#### Participants

We recruited 105 German and 87 Japanese students. However, because some participants failed to report a situation in which they were strongly rejected, the final sample consisted of 92 German (77 women, *M_age_* = 21.64, SD = 2.45) and 80 Japanese students (47 women, *M_age_* = 19.87, SD = 0.87). Although the German sample consisted of more female participants than the Japanese sample, *χ*^2^(2, *N* = 172) = 13.64, *p* = 0.001, a preliminary analysis showed that there were no gender differences in acceptance [*t*(170) = 0.58, *p* = 0.561] or personal choice demand [*t*(170) = −0.73, *p* = 0.464]. Gender was thus not included in the following analyses. Accordingly, data from 81 participants in the social rejection condition (43 Germans and 38 Japanese) and 91 participants in the control condition (49 Germans and 42 Japanese) were analyzed. Based on past research showing a main effect of manipulated social rejection (*d*s = 0.35 and 0.45 in Pickett et al., 2004; *d* = 0.67 in Knowles and Gardner, 2008), we assumed a medium effect size (*d* = 0.50), set the desired power to 0.80, and estimated that the sample size was 63 for each condition.

#### Procedure

We adopted a priming method which successfully manipulated social rejection in previous studies (Pickett et al., 2004; Knowles and Gardner, 2008) and asked half of the participants to recall a situation in which they were strongly rejected, while the other half of the participants recalled their walk to campus that day. Next, participants answered to the three scenarios of Studies 1 and 2 (slightly adjusted to fit students’ daily life experiences) and indicated how likely they would be to accept this vicarious choice or demand personal choice. Finally, they answered demographic questions and were rewarded with 700 Yen or the equivalent of 6 Euros, respectively.

### Results

**Figure 5:**
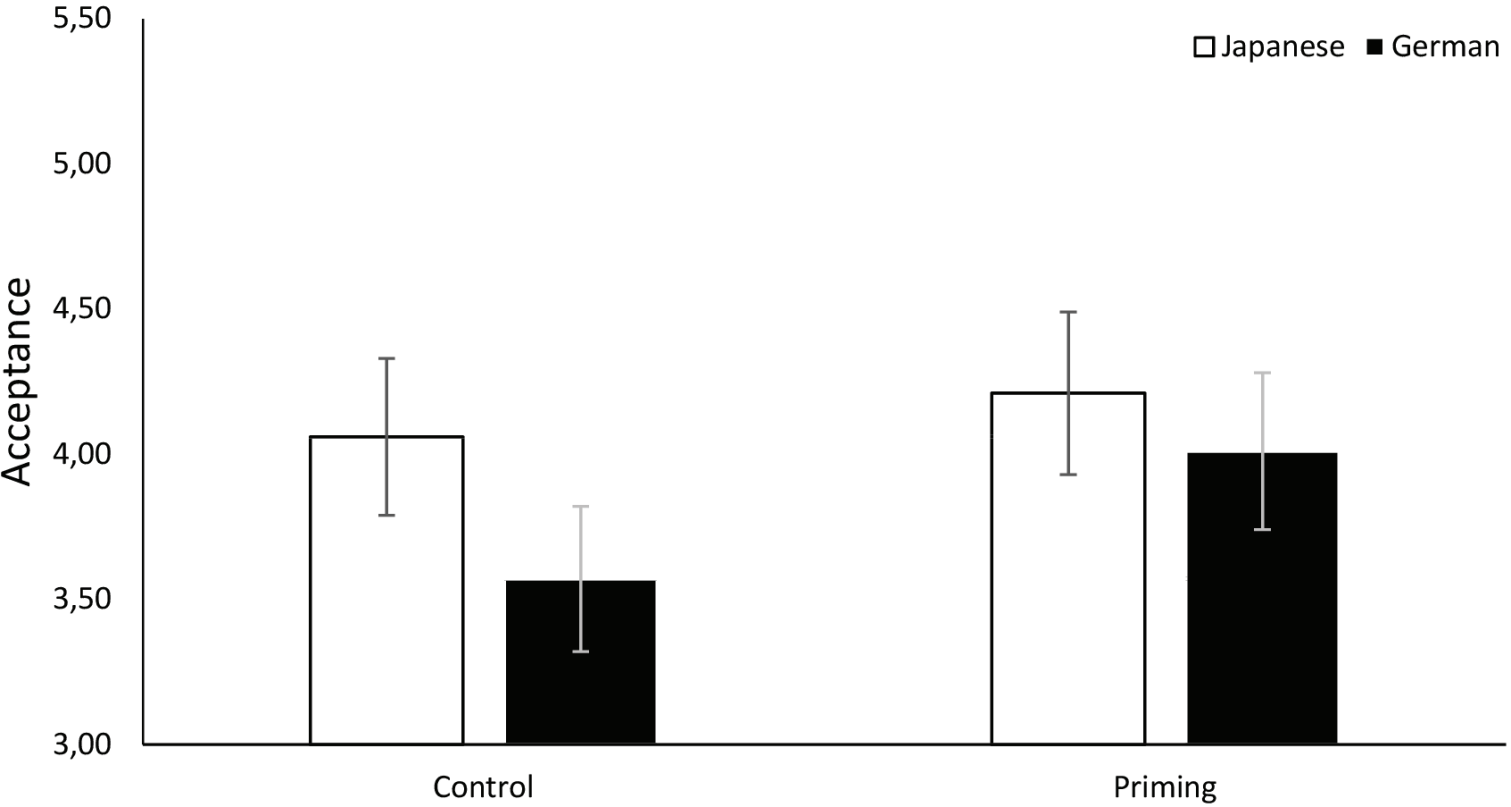
Cultural and group differences in acceptance of vicarious choice in Study 3. Error bars show 95% confidence intervals.

**Figure 6:**
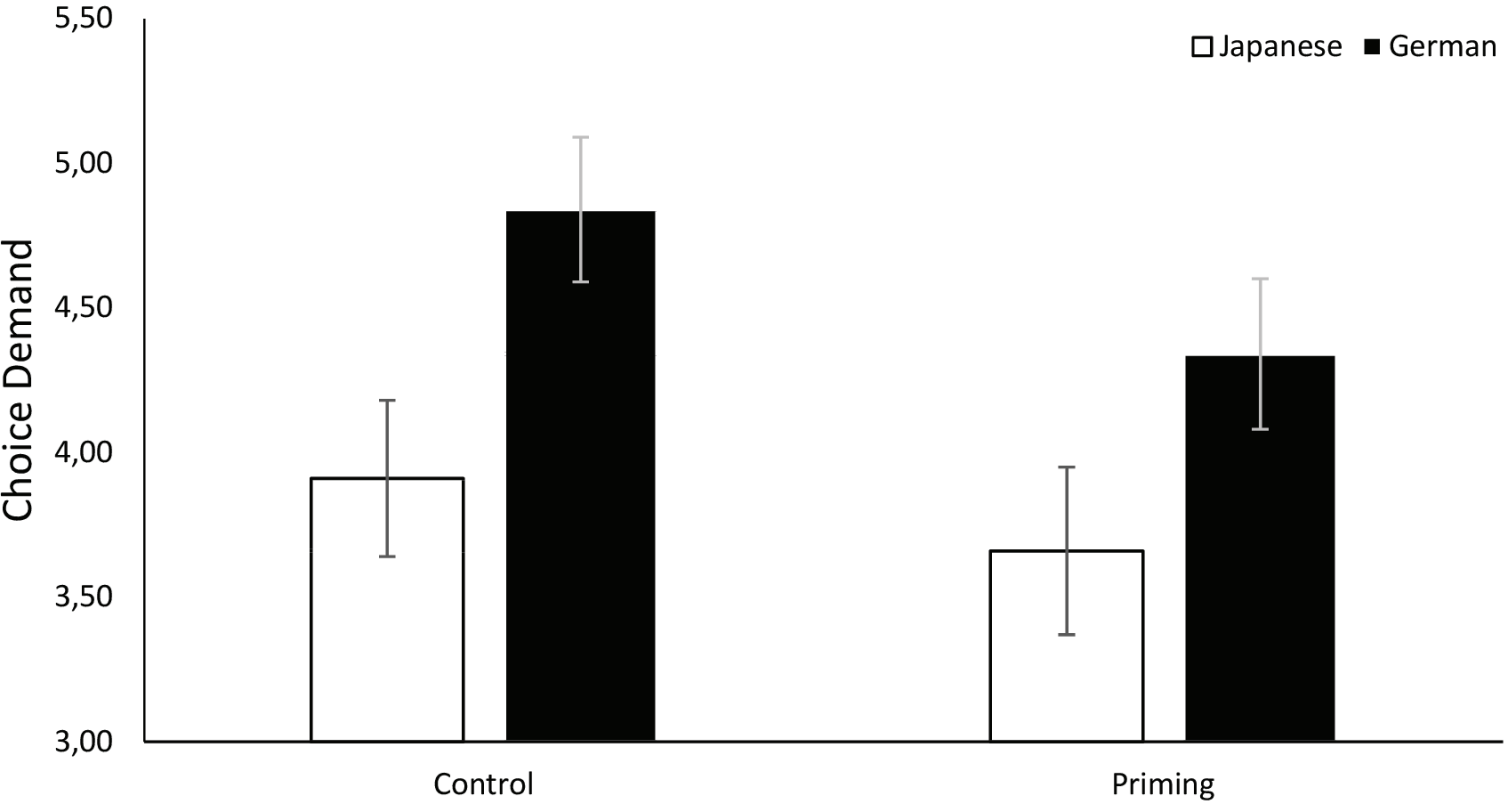
Cultural and group differences in choice demand as a reaction to vicarious choice in Study 3. Error bars show 95% confidence intervals.

As in Studies 1 and 2, we calculated participants’ likeliness to accept vicarious choice (*α*_Ger_ = 0.69, *α*_Jap_ = 0.74) and demand personal choice (*α*_Ger_ = 0.52, *α*_Jap_ = 0.62) over the scenarios. We ran two ANOVAs to test whether culture and priming condition had an influence on acceptance and choice demand, respectively. Regarding acceptance, Japanese students (*M* = 4.13, SD = 0.91) were significantly more likely than German students (*M* = 3.78, SD = 0.89) to accept the choices on their behalf, *F*(1, 168) = 6.42, *p* = 0.012, ${\eta^{2}}_{p}$ = 0.037. Importantly, regardless of culture, participants primed with sensitivity toward social rejection (*M* = 4.10, SD = 0.86) were more likely to accept the ingroup member’s choice than the control group [*M* = 3.80, SD = 0.94, *F*(1, 168) = 4.60, *p* = 0.033, ${\eta^{2}}_{p}$ = 0.027]. The interaction of priming and culture was not significant, *F*(1, 168) = 1.13, *p* = 0.289. Similarly, German students (*M* = 4.61, SD = 0.86) were more likely to demand personal choice than Japanese students [*M* = 3.79, SD = 0.98, *F*(1, 168) = 34.81, *p* < 0.001, ${\eta^{2}}_{p}$ = 0.172]. Furthermore, as predicted, rejection-primed participants (*M* = 4.02, SD = 0.98) were less likely to demand personal choice than the control group [*M* = 4.41, SD = 0.99, *F*(1, 168) = 7.45, *p* = 0.007, ${\eta^{2}}_{p}$ = 0.042]. The interaction was not significant, *F*(1, 168) = 0.76, *p* = 0.385. The results are illustrated in Figures 5, 6.

## General Discussion

Aligning with previous findings, over the studies presented here, Germans indicated to be likely to demand personal choice when a group member had chosen vicariously, while Japanese were found to likely accept vicarious choices. This indicates that Westerners likely perceive vicarious choice as a threat to their rather independently oriented selves, thereby promoting reactance. However, the emphasis on interdependence would lead East Asians to avoid social rejection by meeting others’ intentions and expectations. Hence, the mechanism behind cultural variation in choice seems to be related to the extent to which people are motivated to avoid social disapproval.

This research investigated a specific form of interpersonal choice: psychological consequences of situations in which one group member decides for the entire ingroup without consulting its individual members. This is unique, as previous studies (e.g., Iyengar and Lepper, 1999; Hoshino-Browne et al., 2005; Savani et al., 2008) largely ignored group processes. The focus on groups, however, allowed us to test whether previous findings extent to this very common form of vicarious choice, in which group pressure can be anticipated and perceived. Despite this group pressure, our German participants indicated that they would reject vicarious choices and thereby risk negative social consequences. This gives an idea about the strength of German people’s desire for personal choice. In addition, the results suggest that cultural differences in reactions to an ingroup member’s vicarious choice are not limited to a closely related other like a mother or best friend. Rather, they would extent to colleagues, who are ingroup members but not as closely related.

Moreover, focusing on rejection avoidance, these studies contribute to the literature by partly revealing the mechanism behind cultural differences. Elucidating the mechanism behind cultural differences contributes greatly to the field and advances the knowledge of culture and choice by explaining existing differences rather than merely describing them. In addition, identifying the driving motivations behind variation in behaviors informs psychology in general about the processes underlying individual conducts (Heine and Norenzayan, 2006). Our findings would suggest that interdependently oriented Japanese people are acceptant of choices that imply others’ expectations because falling short of these expectations would result in social rejection or exclusion. In contrast, independently oriented German people were not concerned about social rejection as much and were, therefore, more likely to reject vicarious choices and demand personal choice in order to regain their sense of autonomy. While individual differences in rejection avoidance tendencies affected the likeliness to accept vicarious choice in Japanese and German participants, rejection avoidance did not affect how likely Japanese participants in Study 2 were to demand personal choice. A possible explanation for this is that the norm of not standing out would be so strong that it covered the effect of rejection avoidance at the individual level. However, when we temporarily made rejection avoidance salient with a priming method in Study 3, German and Japanese participants’ tendency to demand personal choice decreased.

The finding that the described vicarious choice situations occur more frequently in Japan than in Germany gives some insight as to the instantiation of cultural differences in daily life. It supports the claim that sociocultural contexts affect individuals by providing them with particular kinds of regularly encountered situations, and the experiences in these socioculturally shaped situations lead to habitual ways of thinking about oneself and the world (Kitayama et al., 1997). As sociocultural contexts foster specific situations that demand specific behaviors and ways of being, individuals learn to construct themselves in order to match these sociocultural expectations. If so, frequently encountered situations might have shaped individuals’ understandings of the self and agency, thereby (unconsciously) advising them on either emphasizing personal, autonomous choice or social connectedness and conformity.

Whereas previous findings were based on studies conducted primarily with North American samples, we examined German people’s behavior and found that they responded negatively to vicarious choice. This is in line with previous findings illustrating that compared to East Asians, Germans considered recommendations by ingroup members less in their workplace choice (Eisen et al., 2016) and that Western Europeans showed strong psychological reactance when they had to give up their personal freedoms (Jonas et al., 2009; Graupmann et al., 2012). Although some previous findings proposed differences in the emphasis on individual achievement and self-promotion between North Americans and Western Europeans (Kitayama et al., 2009), the findings related to choice and agency would not suggest differences between these cultures in the tendency to condemn social influence. However, as this study does not include a North American sample, a direct comparison remains to be addressed in future research.

In our studies, we used vicarious choice stimuli that are likely to happen in everyday life. While we eliminated concerns that culturally different reactions are only side effects of these situations occurring more or less frequent in the two cultures (Study 1a), our findings can be generalized only to everyday choice situations and not to more consequential choices. It is possible that Japanese people are more likely to demand personal choice if the decision is important to them, or alternatively, that increased importance makes them even more likely to reflect upon social approval and accept choices on their behalf. Similarly, more consequential decisions might lead Germans to incorporate the social context more strongly or to be even more likely to decide merely based upon their own preferences. Indeed, previous research suggested that the importance of a decision is a relevant factor to consider (Savani et al., 2010; Li et al., 2014). Furthermore, our scenarios are all work-related and included diverse vicarious choices. Choosing work-related contexts enabled us to create scenarios in which an equal-status ingroup member chose vicariously. However, it is possible that the differences observed pertain to norms about work settings in particular, as opposed to more general cultural differences. In addition, the scenarios described situations in which someone chooses food on behalf of the group, situations in which someone chooses which task each team member has to accomplish, and situations in which a team member responds on behalf of the whole group. This is a very wide understanding of vicarious choice and goes beyond the common definition of choice. Future research needs to explore the generalizability of our findings.

Despite providing insights into the mechanism behind culturally diverse reactions to vicarious choice, rejection avoidance tendencies cannot fully explain sociocultural differences. Additional testing for mediators, such as self-esteem (Heine et al., 1999; Schmitt and Allik, 2005), self-monitoring (Gudykunst et al., 1989), relationship and group-based trust (Yuki et al., 2005) will be necessary to specify the precise factors and their interactions to completely understand the underlying mechanism behind diverse reactions to choice situations.

Another future research direction could be to examine more automatic and unconscious responses to vicarious choice and to investigate its neural mechanism. For instance, given that the dorsal anterior cingulate cortex (dACC) plays a crucial role in the detection of a behavioral conflict, previous research found that cognitive dissonance, particularly conflict evoked by difficult choice, is linked to strong activation of dACC (Kitayama et al., 2013). If Westerners evaluate the group member who chooses vicariously more negatively than East Asians, they might feel that the denial of personal choice caused by the group member’s vicarious choice causes a conflict. If so, the cultural differences in response to vicarious choice would be reflected as cultural differences in activation of dACC.

Finally, it is important to explore whether cultural differences in reaction to vicarious choice mediated by rejection avoidance can be observed even in children. Given that Iyengar and Lepper (1999) tested children ranging in age from 7 to 9 years and found cultural differences in intrinsic motivation toward tasks chosen by either children themselves or others, future work is needed to test children in elementary schools and investigate whether socialization may impact on reaction to vicarious choice as well as rejection avoidance across cultures. This investigation will contribute to our understanding of how children learn and acquire culturally proper forms of choice through socialization.

To conclude, the present research adds to previous evidence on culture and choice by showing that responses to vicarious choice differ across cultures. It also provided the first evidence that the cultural influence on responses to vicarious choice can be explained by cultural differences in rejection avoidance. This evidence for the mechanism that underlies responses to vicarious choice has implications for fields such as marketing and politics particularly in the globalizing world today where people with different cultural backgrounds are urged to work together as a group. Also, the present research presents interesting questions, which should be addressed in future research. We believe that additional insight provided by further investigations suggested in the present research will enhance our understanding of cultural mechanisms behind responses to vicarious choice.

## Ethics Statement

The study was reviewed and approved by the Experimental Research Ethics Committee at the Graduate School of Humanities, Kobe University. Participants provided written informed consent at the beginning of the study. All responses were confidential.

## Author Contributions

CE and KI designed the research. CE and KI performed the research. CE analyzed the data. CE and KI wrote the paper.

### Conflict of Interest Statement

The authors declare that the research was conducted in the absence of any commercial or financial relationships that could be constructed as a potential conflict of interest.

## Footnotes

^1^We admit that dominant can also be seen as a positive feature of a person in some contexts. However, we do not think that this context provokes positive associations with someone who is dominant and therefore treat dominant as a negative evaluation of the chooser.

^2^Please note that when we ran the analyses without this covariate, the results were the same. For acceptance: *t*(207) = 5.89, *p* < 0.001; for choice demand: *t*(207) = −7.74, *p* < 0.001; for positive evaluation: *t*(207) = 5.11, *p* < 0.001; for negative evaluation: *t*(207) = −5.52, *p* < 0.001.

^3^Please note that when we ran the analyses without this covariate, the results were the same. For acceptance: *t*(208) = 6.26, *p* < 0.001; for choice demand: *t*(208) = −7.13, *p* < 0.001; for positive evaluation: *t*(208) = 4.18, *p* < 0.001; for negative evaluation: *t*(208) = −2.14, *p* < 0.05.

^4^We also measured the other three facets of independence and interdependence proposed by Hashimoto and Yamagishi. Compared to the Japanese sample, the German sample indicated higher mean values in engaging independence (= self-expression; *M*_Ger_ = 5.32, *SD*_Ger_ = 1.15, *M*_Jap_ = 4.09, *SD*_Jap_ = 1.09, *t*(430) = 11.53, *p* < 0.001), engaging interdependence (= harmony seeking; *M*_Ger_ = 5.42, *SD*_Ger_ = 1.02, *M*_Jap_ = 4.66, *SD*_Jap_ = 0.97, *t*(430) = 7.94, *p* < 0.001), and disengaging independence (= uniqueness; *M*_Ger_ = 4.94, *SD*_Ger_ = 0.95, *M*_Jap_ = 4.28, *SD*_Jap_ = 0.92, *t*(430) = 7.36, *p* < 0.001). Simple regression analyses revealed that rejection avoidance (*b* = 0.44, *SE* = 0.04, *p* < 0.001), self-expression (*b* = −0.38, *SE* = 0.04, *p* < 0.001), and uniqueness (*b* = −0.28, *SE* = 0.06, *p* < 0.001) significantly predicted acceptance, whereas harmony seeking (*b* = 0.08, *SE* = 0.06, *p* = 0.153) did not. Concerning personal choice demand, rejection avoidance (*b* = −0.32, *SE* = 0.04, *p* < 0.001), self-expression (*b* = 0.61, *SE* = 0.04, *p* < 0.001), harmony seeking (*b* = 0.27, *SE* = 0.05, *p* < 0.001), and uniqueness (*b* = 0.56, *SE* = 0.05, *p* < 0.001) significantly predicted choice demand. Although other facets of independence and interdependence as proposed by Hashimoto and Yamagishi had effects on reactions to vicarious choice, this paper focuses on the effect of rejection avoidance, given that researchers have paid little attention to the effect of this concept across cultures.
